# Hepatitis B, Needle Stick and Medical Workers

**DOI:** 10.4103/0974-777X.68543

**Published:** 2010

**Authors:** Viroj Wiwanitkit

**Affiliations:** *Wiwanitkit House, Bangkhae, Bangkok, Thailand - 10160*

Sir,

I read with great interest the recent publication by Yacoub *et al*. on needle stick among healthcare workers (HCWs).[1] Yacoub *et al*. concluded that “HCWs at Aleppo University hospitals are frequently exposed to blood-borne infections. Precautions to avoid, and protection from, needle stick infections (NSIs) are important in preventing infection of HCWs. Education about the transmission of blood-borne infections, vaccination and post-exposure prophylaxis must be implemented and strictly monitored.”[1] Indeed, the needle stick is a very important route of transmission of blood-borne infectious diseases to the HCWs. As hepatitis B is an important blood-borne viral infection, it is necessary to focus on the needle stick if disease control procedures among HCWs are to be performed. However, there are some facts that need to be addressed. First, among the HCWs in developing countries, there is usually a high prevalence of hepatitis B infection right from their young ages. Wiwanitkit studied medical students and found that many students already had hepatitis B infection before entering into the medical education system.[2] Hence implementation of any program must be done as early as possible among the youngest group of medical personnel. Second, in this work, there might have been some problems due to the nature of retrospective study. Some workers might have forgotten the episode or not reported in the incident-report system. Also, some might have disguised their previous hepatitis B serostatus. Wiwanitkit reported the findings on the subjects who had previously got done a blood test and knew their serostatus: “(1.9%) who said they knew their status declined to give any more details.”[2]

There might have been important technical problems in the study by Yacoub *et al*.[1]
